# Surnames and Y-Chromosomal Markers Reveal Low Relationships in Southern Spain

**DOI:** 10.1371/journal.pone.0123098

**Published:** 2015-04-10

**Authors:** Rosario Calderón, Candela L. Hernández, Pedro Cuesta, Jean Michel Dugoujon

**Affiliations:** 1 Departamento de Zoología y Antropología Física, Facultad de Biología, Universidad Complutense, Madrid, Spain; 2 Centro de Proceso de Datos, Universidad Complutense, Madrid, Spain; 3 CNRS UMR 5288 Laboratoire d’Anthropologie Moléculaire et d’Imagerie de Synthèse (AMIS), Université Paul Sabatier Toulouse III, Toulouse, France; University of Florence, ITALY

## Abstract

A sample of 416 males from western and eastern Andalusia has been jointly analyzed for surnames and Y-chromosome haplogroups and haplotypes. The observed number of different surnames was 222 (353 when the second surname of the Spanish system of naming is considered). The great majority of recorded surnames have a Castilian-Leonese origin, while Catalan or Basque surnames have not been found. A few Arab-related surnames appear but none discernible of Sephardic-Jewish descent. Low correlation among surnames with different population frequencies and Y-chromosome markers, at different levels of genetic resolution, has been observed in Andalusia. This finding could be explained mainly by the very low rate of monophyletic surnames because of the historical process of surname ascription and the resulting high frequencies of the most common Spanish surnames. The introduction of surnames in Spain during the Middle Ages coincided with Reconquest of the territories under Islamic rule, and Muslims and Jews progressively adopted the present male line surname system. Sampled surnames and Y-chromosome lineages fit well a power-law distribution and observed isonymy is very close to that of the general population. Besides, our data and results show that the reliability of the isonymy method should be questioned because of the high rate of polyphyletic surnames, even in small geographic regions and autochthonous populations. Random isonymy would be consistently dependent of the most common surname frequencies in the population.

## Introduction

To achieve a refined knowledge of the human population structure and its reconstruction is advisable to apply different but complementary knowledge bases, which are not only strictly biological. This multidisciplinary approach seeks to reconcile and interpret insights derived from historical events, population dynamics including demographic characteristics, cultural behaviours (mating patterns), and geography with expected strong effects upon extant gene pool.

Surnames, considered key cultural markers, are being used to further investigate and enhance the genetic signals of population structure when analysing genetic data [1,2]. In most western societies, surnames are transmitted through the male line and they are inherited as alleles of a gene [3,4]. Thus, their transmission should closely match that of DNA of the non-recombining region of the Y-chromosome (NRY). This co-inheritance, together with the low cost and ease of collecting and analyzing surname samples, has made surname studies to deal with a great variety of questions and to achieve a wide space within the fields of Anthropology, Population Genetics, Forensic Genetics, Genealogical Genetics, Epidemiology among others [2,5–12]. Nevertheless, other surname characteristics make its use, as a proxy of Y chromosome (Y-C) lineages, be a “crude way to study human inbreeding and migration” [13].

The short evolutionary time of the generalized use of surnames in Europe, which started during Middle Ages, polyphyletism, illegitimacies, adoptions and changes of surnames from their original form (e.g. grammar spellings) all have weakened the strength of relationships between surnames and Y-C lineages. In the vast majority of the countries, the amount of recorded surnames are dozens of thousands, which have represented in turn several orders of magnitude greater than the observed number of specific Y-C haplogroups, and even haplotypes, with the usual number of screened SNPs or STRs, respectively. We will check these points, among others, by analyzing the distributions of surnames and Y-chromosome lineages in a large sample of Andalusian males.

In consequence, those expected frequency relationships have to be assumed as very dissimilar. To take just one example. The most common surname in Spain is *García* (~3%) while broad groups of Y-chromosomes among western Europeans carry the haplogroup R1b1a2-M269 (60–80%) [14–18]. That disparity between proportions would imply that many different surnames would share the same Y-chromosome haplogroup, being presumably common paternal lineages in the population scarcely discriminating in correlations. A stronger link between surnames and Y-C lineages seems more probable for very rare surnames [1]. However, uncommon surnames only account for a very small portion of the population, and as a result, they would have a reduced interest in population genetics studies as well as for epidemiologic, forensic or investigative analyses.

In Spain, there are 75,855 different surnames with more than 19 occurrences recorded in the 2012 Spanish census (*Instituto Nacional de Estadística*, INE, www.ine.es/). Nevertheless, the monumental work of 88 volumes of García-Carraffa and García-Carraffa [19] entitled “*Diccionario heráldico y genealógico de apellidos españoles y americanos*”–“*Heraldic and Genealogical Dictionary of Spanish and American Surnames*”—only describes ~15,000 surnames. In Spain, surnames begun to be adopted among Christian people in the 10^th^ century but they were not widely spread among population until the 12^th^ century. Formal and rigorous rules on surnames were not imposed, however, until the Council of Trent (1545–1563) [20]. The surname introduction in Spain during the Middle Ages coincided with the Reconquest of the territories under Islamic rule. During this long-term process, Muslims and Jews were progressively adopting Christian’s surnames as well as the male line surname system. Later, during the Spanish colonization of America and other Asian regions, like the Philippines, most of native people also adopted Spanish surnames, a process that increased considerably those discrepancies between Spanish surnames and Y-C lineages. Although different languages have been historically spoken in the Iberian Peninsula—Castilian, Portuguese, Galician, Basque, Catalan, Arab, and Hebrew—thus showing distinguishable surnames, it is worth pointing out that most of the current Spanish population is identified by surnames of Castilian-Leonese origin, which are remarkably predominant in all Spanish provinces.

The most common Spanish surnames are characterized by high frequencies in relation to other European countries. In this line, the most frequent surnames in Spain, United Kingdom, France and Italy are *García*, *Smith*, *Martin* and *Rossi*, with frequencies of 3.2%, 0.9%, 0.5% and 0.4%, respectively. However, in some Asian countries, such as China and Korea, frequencies of most common surnames are even higher than those registered in Spain. Therefore, by considering the high representation of the Spanish commonest surnames in the population, together with the history of Spanish emigration over the last centuries, some of the most frequent Spanish surnames are included among those more frequent in other countries (e.g. France and USA).

Some papers aimed to analyze relationships between surnames and Y-C markers (lineages) have been recently published with most of data rising from western Europe (e.g. United Kingdom and Ireland) [1,10,21–26]. In most of these surveys, surnames were firstly selected according to their population frequencies and linguistic characteristics, and the strength of their association with Y-C markers analyzed. By using this procedure, surname samples are biased in relation to local, regional or national populations. Indeed, most of results evidence that each surname is generally related to several Y-C lineages or that each lineage is related to several surnames. Both British and Irish surnames show that relationships between surnames and patrilineal lineages are mediated through the demographic histories of their populations [22].

This paper is aimed at the evaluation of the extent of relationships between Spanish surnames and Y-chromosome markers based on Y-SNP haplogroups and Y-STR haplotypes in a well-defined population sample consisting of unrelated males with family origins in western and eastern Andalusia (southern Spain). We further test whether the used sample size represents adequately the surname structure in the region, and we discuss the reliability of the isonymy method, comparing surname and Y-C lineage distributions, as a proxy of population inbreeding.

## Material and Methods

### Population sampling and population samples

**Fig 1:**
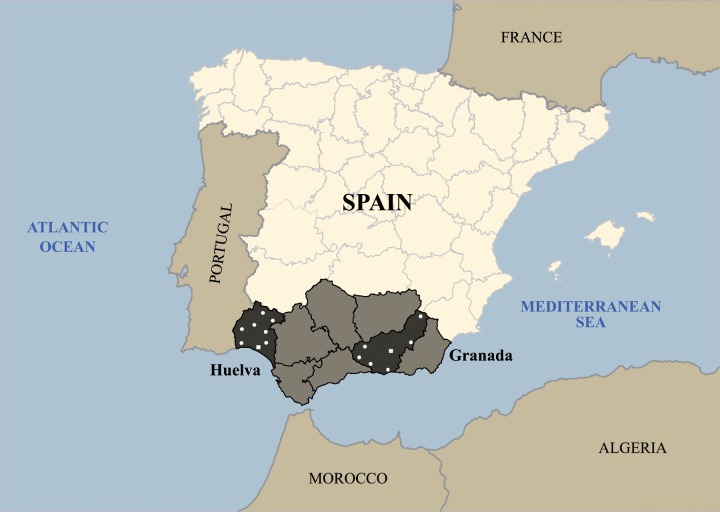
Map of the Iberian Peninsula with Andalusia region. The study provinces of Huelva and Granada are showed in dark color. Locations where blood samples were collected are indicated by white dots and the capital cities of the two provinces are denoted with a white square.

Sampling strategy was planned by members of this team and details of this step have been reported previously [27–31]. Western and eastern Andalusians from Huelva and Granada provinces, respectively, were selected and reasons for this decision are described in the above-mentioned references. For collection of biological samples, localities were chosen following demographic stability and historical criteria. Fig 1 presents the map of Andalusia with the localities where blood samples were collected.

Each participant was personally informed about the aims of the research and asked about his/her first name, first and second surnames, date of birth and birthplace. In order to define “autochthony”, up to third generation, birthplaces of parents and all four grandparents were requested as well. In Spain, and other countries historically and culturally related to it, each individual is identified by two surnames instead of a single one as in the vast majority of countries. Women do not change their surnames when they marry. For both genders, the first surname is his/her father’s first one and the second surname is his/her mother’s first one, which is in turn the first surname of her mother’s father. This important particularity permits to identify surnames two generations backward (the generation of the grandfathers). In the present work, except when specified, we will refer to first surnames in the sample.

The population sample studied here is a subset of a larger one used in our ongoing project on Andalusian population structure, in the frame of the Mediterranean space. Genetic data derived from autosomal and uniparental markers—based on these samples—have already been published [27–33].

### Ethics Statement

Members of this research team, helped by local health workers of the Andalusian Health Service, collected the blood samples from donors after they had given their written informed consent. The Bioethics Committee of the Complutense University of Madrid (Spain) approved the procedures.

### DNA extraction and Y-chromosome DNA analysis

A total of 416 Andalusian healthy, unrelated males were collected and molecularly analyzed [Huelva (*n* = 167) and Granada (*n* = 249)]. Genomic DNA was extracted from total blood (≈5 ml) (taken on EDTA tubes) using standard proteinase-K digestion followed by phenol-chloroform extraction and ethanol precipitation. DNA samples were firstly genotyped for 17 Y-STRs (Short Tandem Repeats or microsatellites) (DYS19, DYS389I, DYS389II, DYS390, DYS391, DYS392, DYS393, DYS438, DYS439, DYS437, DYS448, DYS456, DYS458, DYS635, Y GATA H4, DYS385 a/b) included in AmpFlSTR Yfiler Kit (Life Technologies). Granada sample (*n* = 249) was reduced for technical reasons to 223 individuals when typing the array of microsatellites. Alleles of microsatellites were designated based on the number of variable repeats included [29]. In addition, all Andalusian male samples (*n* = 416) were genotyped for the Y-*Alu* polymorphism (YAP element DYS287), 12f2 polymorphism, microsatellites DYS413 and DYS445 and also for 49 Y-SNPs (Single Nucleotide Polymorphisms). The latter set of markers were characterized using either PCR-RFLP procedure, direct sequencing and/or multiplex typing assays, following a hierarchical order (Calderón et al., manuscript in preparation). For the Y-STR allele nomenclature, we followed the recommendations given by the DNA Commission of the International Society of Forensic Genetics (ISFG) http://www.cstl.nist.gov/strbase. Updated nomenclature to classify Y-DNA chromosome haplogroups and subhaplogroups can be found at http://www.isogg.org/tree. Genotyping and sequencing were carried out in the Laboratory of Molecular Anthropology (Complutense University, Madrid) and the Genomics Unit (UCM-Madrid Science Park).

### Statistical and Genetic Data Analysis

Isonymy stands for mated pairs having the same surname. When pairs are randomly chosen from the population, isonymy, *I* is defined as: *I* = 4*F*_*r*_ = ∑*x*_*i*_^2^ where *F*_*r*_ is the random component of the inbreeding coefficient by isonymy, and *x*_*i*_ is the relative frequency of surname *i*th in the study sample. The formula is based on the assumption than individuals with shared surnames are more closely related than individuals without that condition. Data on surnames of married pairs (marital isonymy) permit, if necessary, the calculation of the non-random component of the inbreeding coefficient, *F*_*n*_. Thus the total inbreeding coefficient, *F* can be expressed as *F* = *F*_*r*_+ (1-*F*_*r*_) *F*_*n*_ (see p.21 in [5]).

When analyzing surname data sets, the term “Occurrence” refers to the number of cases of surname "*i*" in the sample whereas “Abundance” is the number of surnames with "*j*" occurrences.

ARLEQUIN v. 3.5 [34] was used for different purposes: haplogroup (*h*) and haplotype (*H*) diversities; Ewens-Watterson non-parametric tests; population pairwise *F*_*ST*_ between first and second surnames; and mutational steps between Y-C haplotypes. Other data analyses were performed using SPSS v. 22 program (IBM Corporation, New York, USA).

Contingency tables have been used to test if observed values of R1b1a2-M269 lineage is significantly associated with surname occurrences. Data were arranged into six groups in order to avoid classes with low number of cases. The designed interval classes were: 21–8, 7–5, 4, 3, 2 and 1.

The database of the 2010 Spain census (www.ine.es/, accessed 07/12/2011) has been chosen since it would be the less affected by most recent immigration towards Spain. Surnames were coded to anonymize donors. For the Andalusian case, the population frequency of each surname was calculated as the weighted average of the current population sizes in Huelva and Granada provinces.

## Results

In the sample of 416 Andalusian males, 222 different first surnames were observed of which 159 were singletons and the other 63 surnames (≥2, *n* = 257 individuals) ranged in frequencies from 2 to 21 (see S1 Table). The surname with the highest incidence was “*gc”* with 21 bearers (5.05% of the total sample).

**Fig 2:**
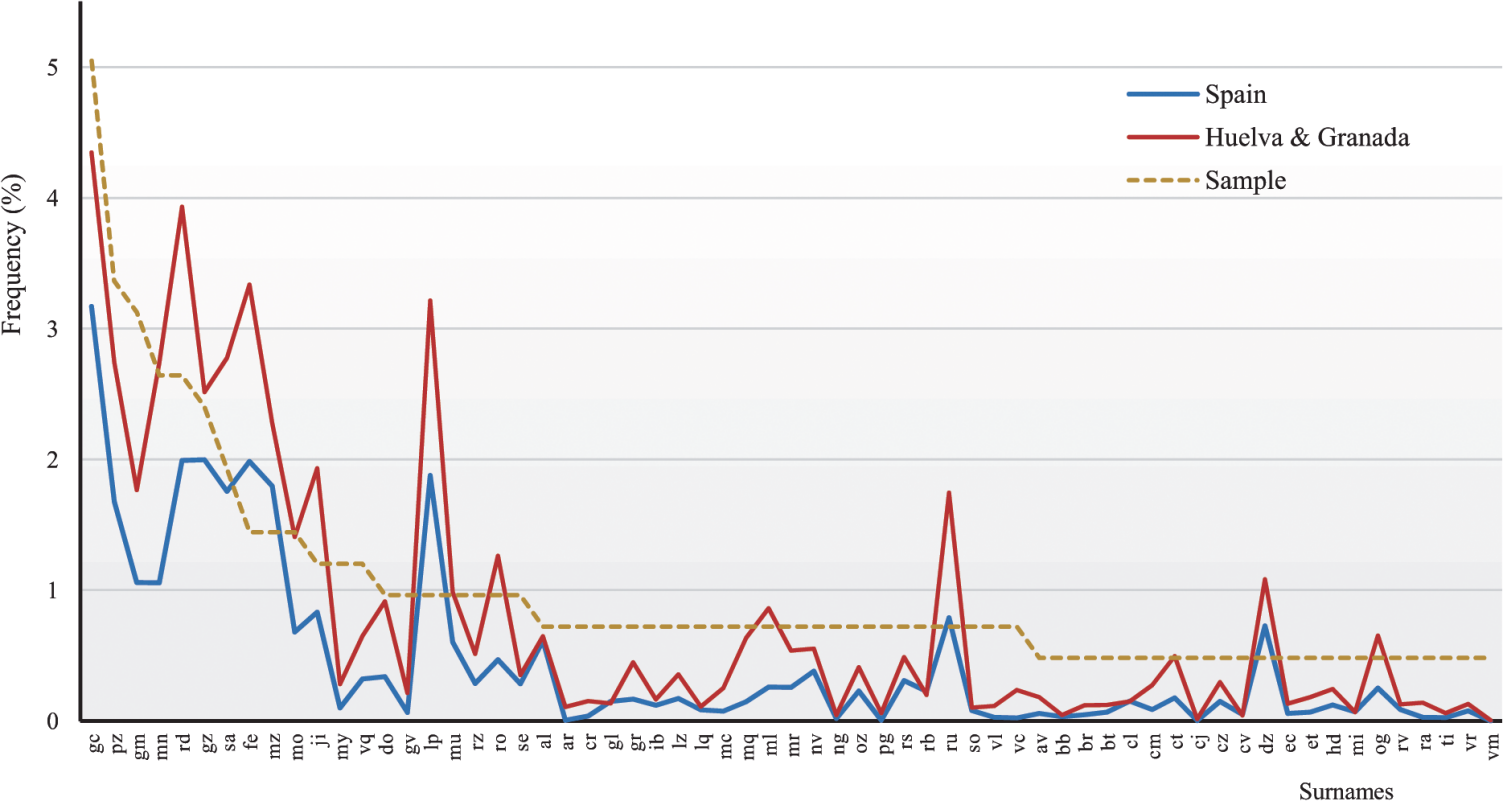
Frequency distributions of the most common Spanish first surnames. Values in the Andalusian sample were compared with those observed in Spain and in the weighted average*of Huelva and Granada provinces (census 2010). *based on current population sizes.

Fig 2 shows the distribution of the 63 non-singleton surnames observed in the sample and their respective values in whole Spain and the weighted average of Huelva and Granada. Interestingly, the three frequency distributions display similar patterns. In a national scale, 26 of those repeated surnames occurred at frequencies lower than 0.001 and 4 surnames: “*ar”*, “*cj”*, “*pg”* and “*vm”* occurred with frequencies <0.0001. In a weighted provincial scale the corresponding figures were 8 (<0.001) and 1 (<0.0001).

**Fig 3:**
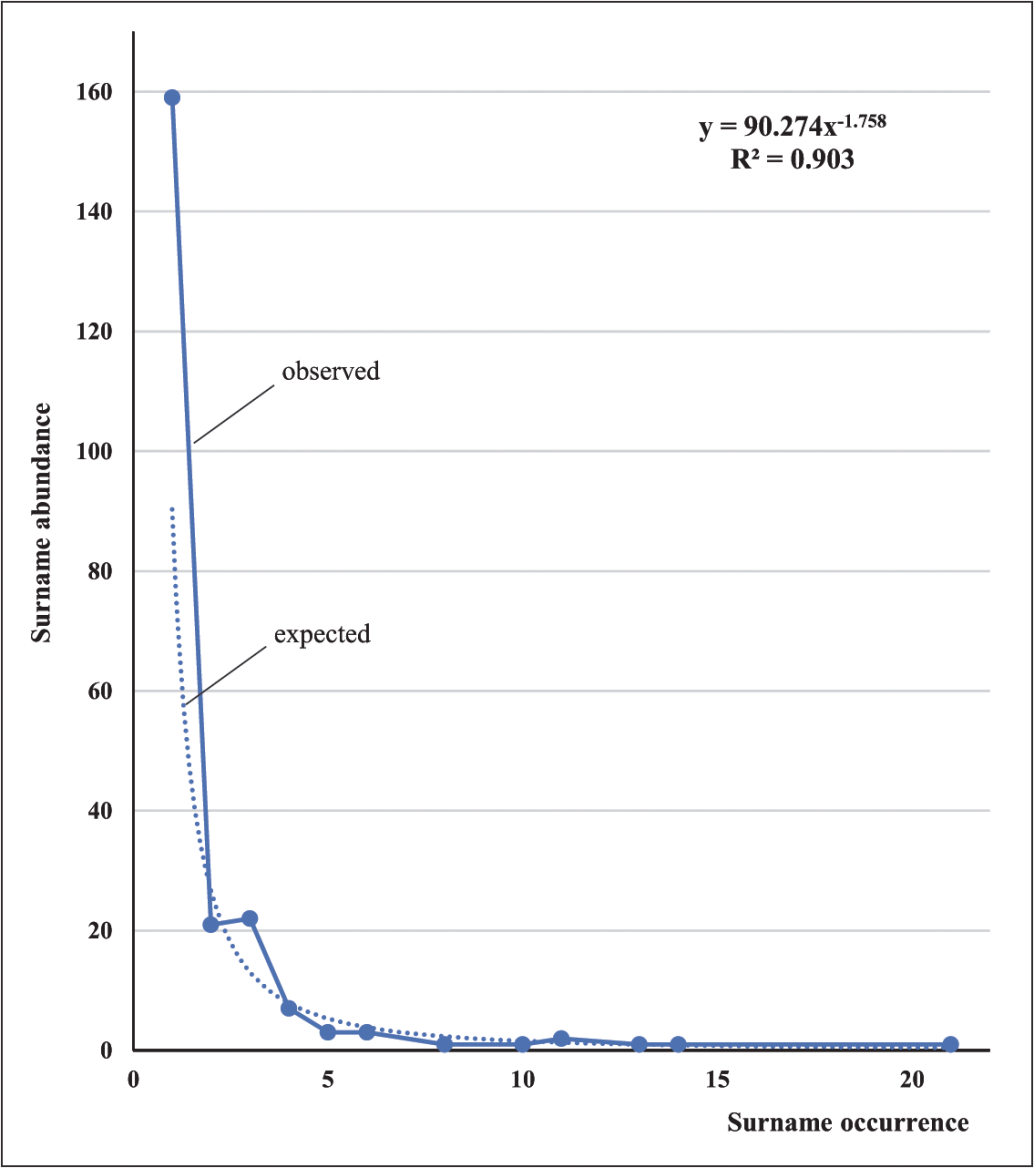
First surname occurrences *versus* abundances distribution in the Andalusian sample. The corresponding expected power law distribution (dashed line) is plotted.

In this study, the plot of occurrence *vs* abundance of first surnames fits well a power-law distribution *y* = 90.27 *x* ^-1.76^, *R* ^2^ = 0.903 (Fig 3). Similar results and fits were drawn for second surnames and when combining first and second surnames. In most human populations, the power-law exponent usually ranges between -2 and -1.5 [35–37]. When there are population admixtures or important presence of surnames of different languages the exponent is near to -1.5.

Table 1 presents computed values of *R* ^*2*^ and power law parameters, Ewens-Watterson test, and population pairwise *F*_*ST*_ between first and second surnames. Comparisons between first (father’s name, or *apellido paterno*) and second surnames (mother’s name, or *apellido materno*) were performed and differences were not statistically significant (*F*_*ST*_ = 0.00014, *P*-value = 0.35 n.s.). This finding suggests that the probability of first and second surnames come from the same population cannot be ruled out. Nevertheless, there are significant discrepancies between the observed surname frequencies and expectation under the Infinite Alleles Model (IAM) for all cases here considered (*P* = 1.000 in the Ewens-Waterson test). The detected deficit of genetic diversity is probably due to a higher observed frequencies than those expected for sharing most common and singleton surnames. A similar effect appears under purifying selection (see p.142 in [38]) and, in the present context, it should be related to *polyphyletism* and/or the recent introduction of surnames.

**Table 1:** Observed surnames in the Andalusian sample.

				*y = Ax* ^*B*^			*Ewens-Watterson test*			
Surnames	*n*	*k*	*Singletons*	A	B	*R* ^*2*^	Obs *F* value	Exp *F* value	*P*-value	*F*_*ST*_(Surn 1 *vs* Surn 2)
***First Surnames***	416	222	159	90.27	-1.758	0.903	0.01126	0.00751	1.000	0.00014^a^
***Second Surnames***	416	212	142	81.18	-1.731	0.885	0.01142	0.00816	1.000	
***First + Second Surnames***	832	353	229	105.13	-1.593	0.881	0.01008	0.00552	1.000	

Power law distributions with the calculated parameters and goodness of fit to the data of surnames as well as their Ewens-Waterson tests are shown. *F*_*ST*_-statistic value for the first *versus* second surnames is provided.

*n*, *sample size*.

*k*, *number of different surnames*.

^*a*^ *P-value n*.*s*. *(>0*.*05)*.

**Fig 4:**
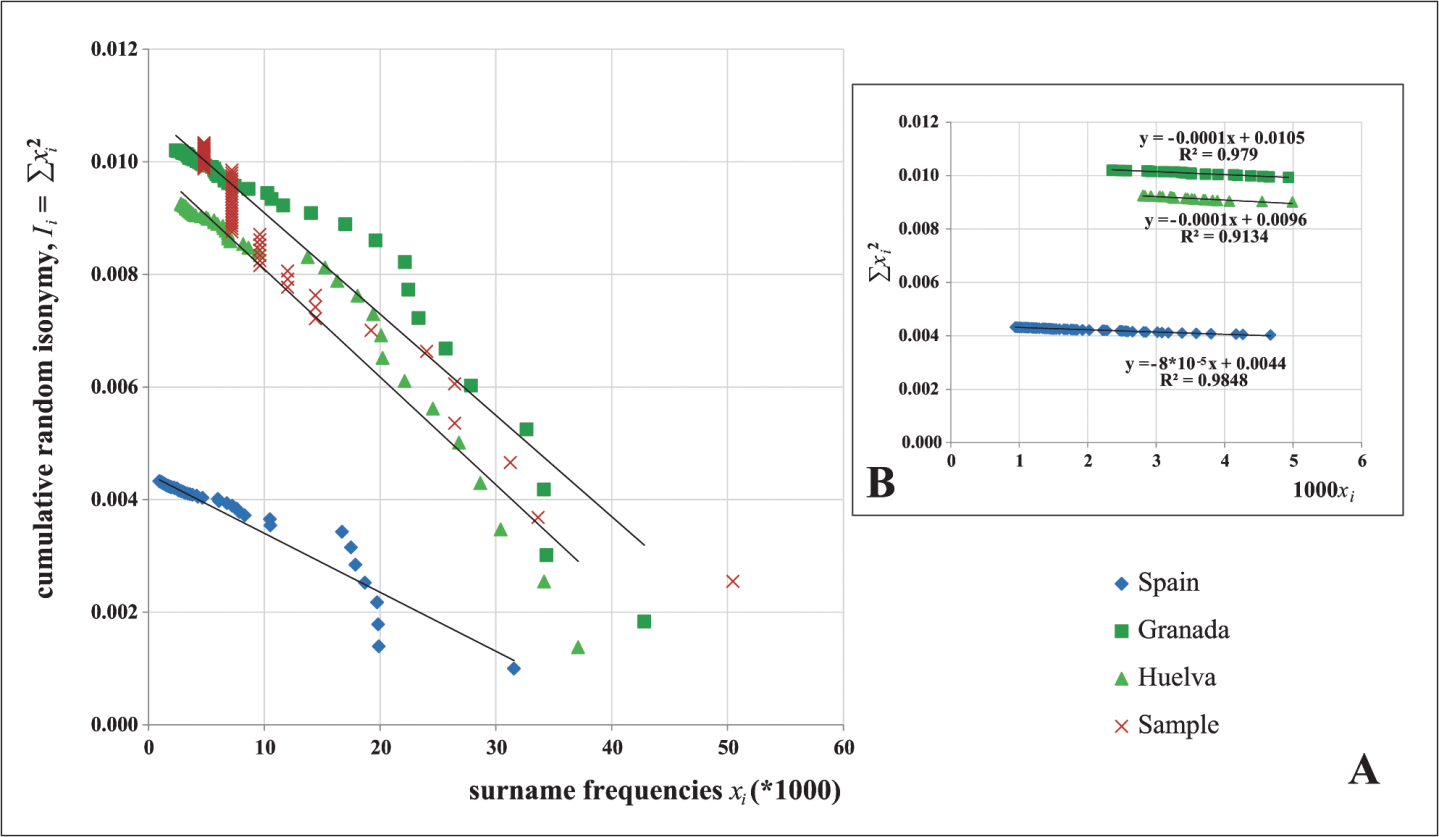
Cumulative random isonymy *I*_*i*_ values for surnames with frequencies ≥ *x*_*i*_ versus surname frequencies *x*_*i*_ (*1000). (a) Cumulative random isonymy in the Andalusian sample. For comparisons, random isonymy values based on the most common surnames in the population of Spain (100) and Huelva and Granada provinces (50). (b) Regression analyses for cumulative random isonymy data tails in the populations above indicated have been fitted.

Sample random isonymy of first surnames was *I* = 0.0113. We compare this value with the corresponding estimates for Huelva and Granada provinces and Spain using census data (2010) (www.ine.es/) (see Fig 4A). This official, open-access source of information provides frequencies of the 50 top surnames in each Spanish province and the 100 top surnames for whole Spain. The corresponding random isonymy figures, denoted by *I*_*1*_, and based on these top surnames were: 0.0043 for Spain and 0.0093 and 0.0102 for Huelva and Granada provinces, respectively. Contribution of the other less frequent surnames (from 101th- or 51th-) to the isonymy, denoted by *I*_*2*_, is small. Obviously, *I* = *I*_*1*_ + *I*_*2*_. To compute *I*_*2*_ we can extrapolate the *I*_*1*_ tails based on 100 or 50 top surnames or calculate upper and lower bounds for *I*_*2*_ (see Fig 4B). The upper bound is *I*_*2max*_ = (1-*p*_*l*_)*x*_*l*_ where *p*_*l*_ is the portion of the population which bears the most common provided surnames and *x*_*l*_ is the frequency of the less frequent (101th- or 51th-) among the most common ones in the group; the lower bound is *I*_*2min*_ = (1- *p*_*l*_)/*N* where *N* is the population size. In other words, when all those not considered surnames have a frequency equal to *x*_*l*_ for the upper bound or when all of them are singletons for the lower limit. *I*_*2*_ is generally closer to *I*_*2min*_. From the Spanish census we know that the top 50 surnames accounts for 50% in each two Andalusian provinces (50.02% in Huelva; 50.36% in Granada). In Spain, ~38% of the population carry the 100 most common surnames. By the extrapolation method, random isonymy estimates would be *I* = 0.0044 for Spain, *I* = 0.0096 for Huelva and *I* = 0.0105 for Granada. The respective upper bounds are 0.0044, 0.0107 and 0.0114. For both provinces, these assessments closely agree to those obtained from our sample. Then, it suggests: *i*). A reliable signal of the good fit between size and frequencies of sampled surnames in relation to the populations where bearers of those surnames come from, *ii*). Random isonymy is strongly determined by the frequencies of the most common surnames, which accordingly show a high polyphyletism.

Additional caveats of *I* to estimate random isonymy [the distribution of neutral alleles in a population of haploid individuals is *θ*_*I*_ = *I* ^-1^–1] are that it uses the less informative part of the data. This means it is strongly biased having a mean quadratic error from six to eight times higher than the maximum likelihood estimator ([39], p.112 and 303 in [40]).

### Y-SNP haplogroups and surnames

Haplogroup diversity, *h* across 63 repeated surnames and other related information is provided in S1 Table. The whole-sample *h*-value (included repeated and singleton surnames) was 0.6250±0.0281. As expected, the weight of singleton surnames was higher (*h* = 0.6990±0.0412) than that calculated for repeated surnames (*h* = 0.5787±0.0368).

A number of 27 out of 63 repeated surnames (42.86%) shared identical haplogroups (*h* = 0) and the majority (24/27) were associated to R1b1a2-M269 haplogroup. Cases in which the same surname occurs two or more times within the same haplogroup accounts for 184 out of 257 of which 155 correspond to the abovementioned marker, with peak frequencies in western Europe (60–70%). In the study sample the frequency of R1b1a2-M269 was 62%. Furthermore, a significant relationships between frequency distributions of R1b1a2-M269 and surname occurrences was observed (χ^2^ = 13.49, *df* = 5, *P* = 0.019*). When using Fisher's exact test, the *P*-value was very similar (0.016). The level of significance can be explained by large deviations between observed and expected counts. The categories of cells 4 and 2 (see M & M) with standardized residuals lower than 2.00 were those having a minor contribution to the chi-square (χ^2^) value. Similar statistical analyses for other haplogroups are unfeasible because their low number of cases in the population (≤ 5).

**Fig 5:**
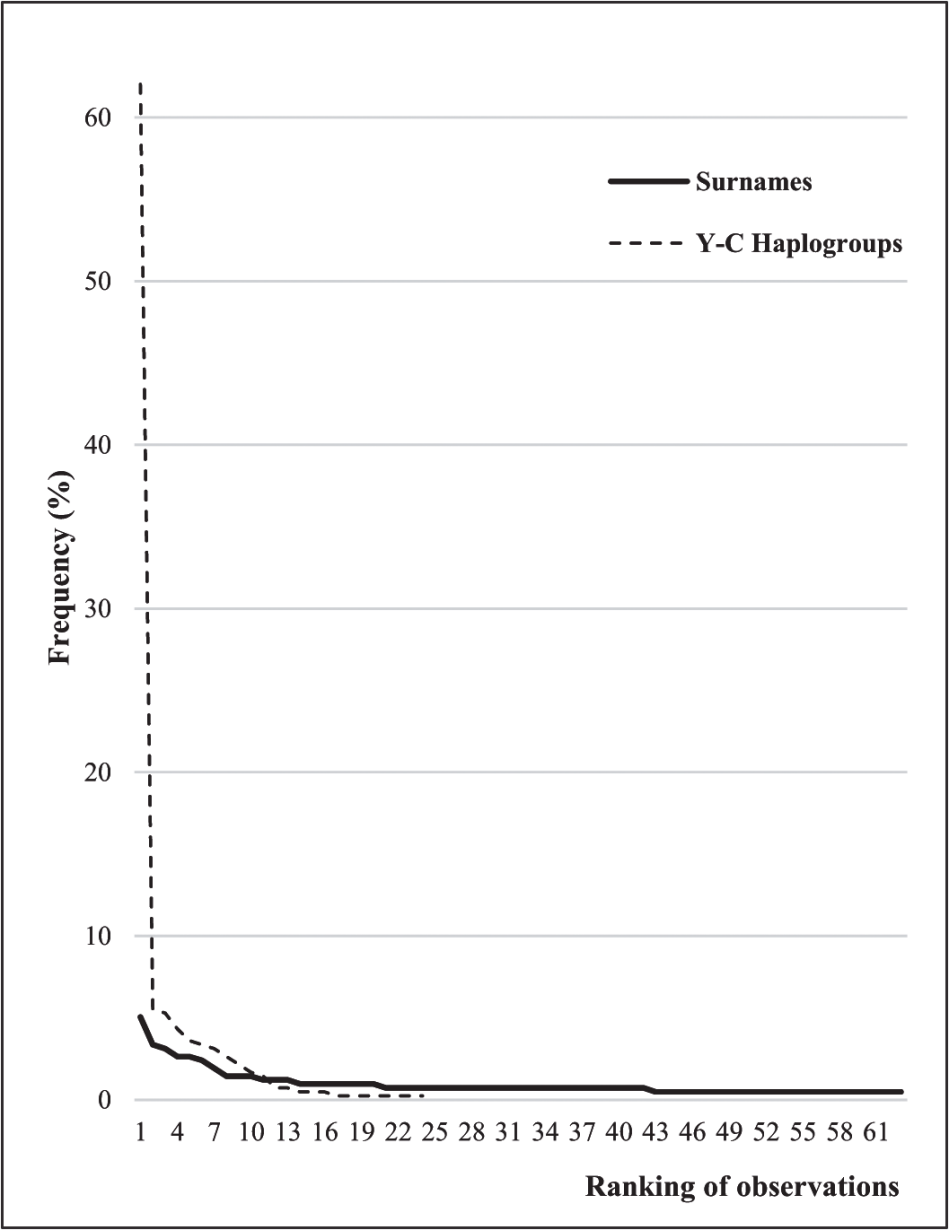
Ranked distributions of Y-chromosome haplogroups and first surnames observed in the Andalusian sample.

Interestingly, we found three surname cases with 3 occurrences each carrying haplogroups E-M81, I1-M253, and J2-M172 respectively. The former lineage reaches very high frequencies in Berbers from northwestern Africa [41] whereas the latter is found mainly in the Fertile Crescent and Mediterranean (southern Europe and northern Africa) [42,43]. The genetic geography of I-M253 reveal maximum frequencies (88–100%) in northern Europe with a decreasing cline from Scandinavia toward the Urals and Atlantic periphery [44]. Other paternal lineages such as E-V13, E-M34, I2-P215, and J1-M267 connected to specific surnames in the study sample are worthwhile standing out. Fig 5 plots the frequency distribution of haplogroups and surnames in the Andalusian sample.

**Fig 6:**
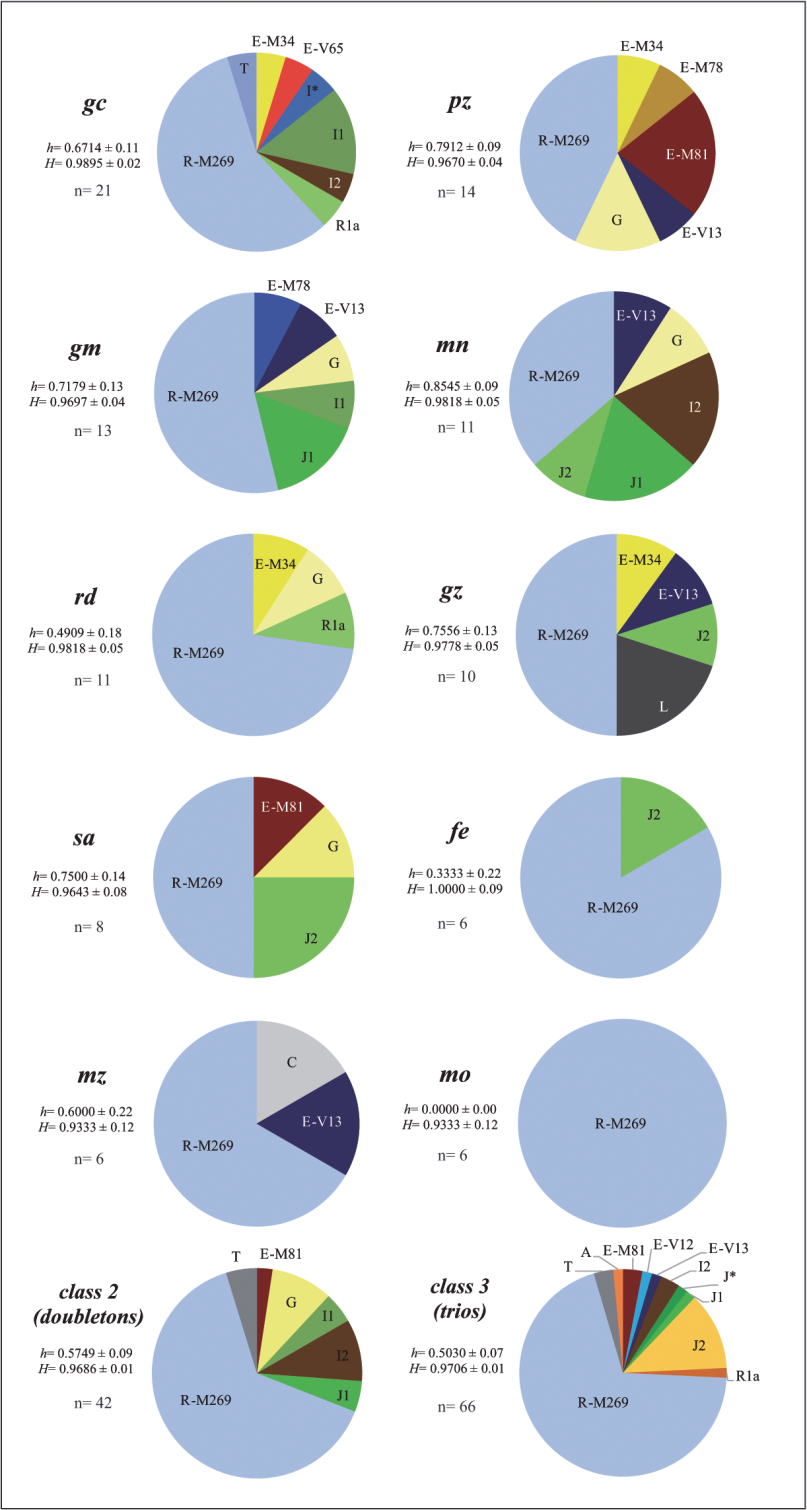
Y-chromosome haplogroup composition in surnames with more than one occurrence in the Andalusian sample. The ten most common surnames and the surname classes with two or three occurrences were represented. For each surname and classes, gene diversity based on haplogroups, *h* and haplotypes, *H* and number of individuals are provided.

Fig 6 shows Y-C haplogroup compositions for the ten most frequent surnames in the sample and for doubleton and trio surnames. The majority of this surname set contains a marked haplogroup diversity (*h*-values: 0.778–0.446) with R1b1a2-M269 being the most common haplogroup in all cases. The presence of other Y-chromosomal haplogroups: E-V13, E-M81, E-M34, J1-M267, J2-M172, I1-M253, I2-P215 and G2a-P15 would be interpreted as a consequence of the genetic history linked to the Iberian Peninsula, especially, to Andalusia, with a long, prevailing contacts with the Mediterranean world. The findings would be showing indeed that a remarkable number of descendants of distant migrants established in the Andalusia territory adopted the most common Spanish surnames.

### Y-STR haplotypes and surnames

Haplotypes based on multiple Y-specific microsatellites represent more sensitive indicators of recent coancestry than haplogroups. They are highly variable and have much lower average population frequencies than do haplogroups, so the chance of sharing is less likely [1].

From the 390 DNA male samples, the number of different haplotypes observed was 184 (7 locus Y-STRs) and 354 (17 locus Y-STRs). As expected, the extent of global haplotype diversity, *H* was particularly high (0.9792±0.0032, 7 Y-STRs; 0.9989±0.0005, 17 Y-STRs) being the mean number of mutational steps (number of loci differences) between haplotypes (7 locus Y-STRs) 3.86±1.96 (3.75±1.89 when only examining the group of repeated surnames).

It has been pointed out that unrelated men sharing surnames were significantly more likely to share haplotypes than are men carrying different surnames [10,23]. Table 2A and 2B present the array of haplotype occurrences in the sample, the number (abundance) of each haplotype class as well as the number of shared (coincidences) surnames-haplotypes. Data shows that: *i*). A great number of haplotypes exhibited the condition of being singletons: 124/390 (31.79%, 7 Y-STRs) and 323/390 (82.82%, 17 Y-STRs), *ii*). There are only a small group of 39 Y-chromosomes (10% of the total) that bearing different surnames share all the same haplotype: **DYS19**(14)-**DYS389I**(13)-**DYS389II**(16)-**DYS390**(24)-**DYS391**(11)-**DYS392**(13)-**DYS393**(13). When examining haplotypes across 17 loci the modal haplotype (*n* = 5) was: **DYS19**(14)-**DYS389I**(13)-**DYS389II**(16)-**DYS390**(24)-**DYS391**(11)-**DYS392**(13)-**DYS393**(13)-**DYS438**(12)-**DYS439**(12)-**DYS437**(15)-**DYS448**(19)-**DYS456**(15)-**DYS458**(15)-**DYS635**(24)-**Y GATA H4**(12)-**DYS385** (11–15) and, *iii*) A low proportion of sharing haplotypes within surnames has been observed [22.18% (57/257 people) 7 Y-STR haplotypes; 11.67% (30/257 people) 17 Y-STR haplotypes].

**Table 2:** Occurrences-Abundances of Y-STR haplotypes and number of first surname—haplotype (*Ht*) coincidences in the Andalusian sample.

***a) 7 locus Y-STR Haplotypes*** ^***1***^						
*Haplotype Occurrences*	*Haplotype Abundances*	*Singletons*	*Doubletons*	*Trios*	*Surname-Ht Coincidences*	*Number of individuals*
1	124	124	-	-	0	124
2	31	52	5	-	10	62
3	13	31	4	-	8	39
4	6	18	3	-	6	24
5	1	3	1	-	2	5
6	2	7	1	1	5	12
7	1	5	1	-	2	7
9	2	15	-	1	3	18
14	1	12	1	-	2	14
23	2	40	3	-	6	46
39	1	26	5	1	13	39
		**333**	**24**	**3**	**57**	**390**
***b) 17 locus Y-STR Haplotypes*** ^***2***^						
*Haplotype Occurrences*	*Haplotype Abundances*	*Singletons*	*Doubletons*	*Trios*	*Surname-Ht Coincidences*	*Number of individuals*
1	323	323	-	-	0	323
2	28	15	13	-	26	56
3	2	2	-	-	0	6
5	1	1	2	-	4	5
		**341**	**15**	**0**	**30**	**390**

^1^Power-law distribution parameters: A = 38.747, B = -1.267; R^2^ = 0.6801.

^2^Power-law distribution parameters: A = 293.28, B = -3.799; R^2^ = 0.9551.

Table 3 shows the most frequent Y-C lineages (haplogroups) reported in this study and the number of shared haplotypes/surnames for each lineage. It is worth pointing out that only 38 individuals (23.03%) out of 165 R-M269 bearers shared identical surnames and haplotypes 7 Y-STR loci (that low proportion is also true for 17 Y-STR loci). In comparative terms, J1-M267 and J2-M172 lineages showed the highest relationships, especially the latter. The presence of J1-M267 sub-haplogroup in Iberia is likely a consequence of Arabic migrant populations that entered the Peninsula during the Islamic expansion. By contrast, the J2-M172 seems to be related to Greek and Phoenicians colonies that were well stablished at least from the first millennium BC in the Peninsula, particularly in littoral Andalusia [28]. Other migrations occurring in ancient times from the Fertile Crescent cannot be discarded. In this line, the higher rates of sharing haplotypes associated to J1 and J2 lineages could be due to polygamy, a marital behaviour that was, and still is, practised culturally by the Arab population.

**Table 3:** First surname-haplotype coincidences within specific haplogroups in the Andalusian sample.

		*Surname-Haplotype Coincidences*			
		*7 Y-STR haplotypes*		*17 Y-STR haplotypes*	
Haplogroup	N	n	%	n	%
**R1b1a2-M269**	165	38	23.03	20	12.12
**I1-M253**	7	2	28.57	2	28.57
**I2-P37**	11	2	18.18	0	0.00
**J1-M267**	9	4	44.44	2	22.22
**J2-M172**	15	9	60.00	4	26.67
**E-V13/A**	2	2	100	2	100
**Total**	257*	**57**	**22.18**	**30**	**11.67**

*Sampled individuals with repeated surnames are 257.

In summary, our study sample shows few concordances between Y-C haplotypes and surnames. False paternities and mutational pressure can cause part of the discrepancies. Mutational events (genetic or linguistic) seem to have a low effect here due to the few generations passed from the introduction of surnames to the present and the rather rigid and perdurable orthography and phonetics of the Spanish language. False paternities would not be relevant here, because the most common haplotypes are mostly composed of different surnames and birthplaces of individuals carrying those haplotypes are different, too. Obviously, false paternity events tend to occur in the same locality or in its proximity.

### Spanish surnames and historic groups

First sampled surnames have been compared with available lists of Spanish surnames of Arab and Jewish origins ([45], http://www.ifmj.org). The former list comprises 1620 Arab surnames and the latter one 5220 Sephardic surnames. Eleven different Spanish surnames with Arab origin were detected (5 first surnames and 11 second ones) all of them singletons in each group. Of the 5 first surnames, two are linked to the Y-chromosome R-M269 haplogroup whereas the rest were associated to E-M81, E-V13 and J2b2-M241 lineages. Thus, some relationships between specific NRY haplogroups and Spanish-Arab surnames with origin in northern Africa and eastern Mediterranean are observed in our Andalusian sample. By contrast, many of those Sephardic surnames are high or moderately frequent in Spain and coincident with those registered in our sample. Since Sephardic surnames are scarcely distinctive, in this case the provided information about the geographic origin on Y-C lineages related to Sephardic-Jewish surnames in our sample is consequently poor.

## Discussion

The present study, mainly designed to evaluate the extent of relationships between surnames and Y chromosome diversity in a random population sample collected from Andalusia region, has evidenced that this association is low, and generally, it decreases when passing from Y-SNP haplogroups to Y-STR haplotypes as well as when the genetic marker resolution increases. Moreover, data does not seem to show signals of a higher number of surname-haplogroup associations in rare than in common surnames, as some researchers have pointed out [1,10]. When the highly represented R-M269 haplogroup is excluded, that conclusion is even more perceptible. The stated inferences could be extrapolated to other Y-C haplogroups in samples of larger sizes.

The observed findings would be mainly explained because of the historical process of surname ascription or adoption, the high level of polyphyletism, and discrepancies between surnames and Y-chromosome lineage distributions in human populations. Main types of surnames such as patronymic (e.g. *Fernández*, which refer to Ferdinand son); toponymic (e.g. *Ávila*, the Castilian city of Ávila, thus indicating a birthplace); occupational (e.g. *Herrero*, meaning blacksmith), and of appearance (e.g. *Rubio*, meaning fair-haired, blond) would not be expected to show higher relationships with specific Y-C lineages than others. Some exceptional cases could be found out in extremely rare surnames in the population.

The observed spectrum of surnames in the study Andalusia sample is supported by history. Castilian-Leonese men mainly carried out the Reconquest of southern Spain from the Muslim rulers, and many of those people resettled there. Leon and Castile regions had a high demographic size in the Middle Ages and played a dominant role during the process of the Reconquest of Spain. Thus, it can explain that the great majority of our sampled surnames have a Castilian-Leonese etymology whereas Galician and Portuguese surnames are occasional, despite that Huelva province is adjoining to Portugal. Only few surnames tracing to an Arab etymology have been detected but it was not the case for Catalan, Basque or even discernible Jewish surnames. Successive historical human movements into Andalusia have not significantly changed this scenario. A curious signal of preferences shown by Berber and Jewish people, by embracing particular Christian surnames, can be exemplified by “*pz”* surname, which just carry either E-M81 or E-M34 paternal haplogroups. Other surname cases reveal once again the complex relationships between Y-C markers and surnames. The toponymic surname “*vc”* refers to a northern Andalusian town with a castle, which dominated and controlled a main road connecting Andalusia and Castile. This castle was defended by a Muslim garrisons in the 12^th^ century. In this survey we have found three carriers of this surname with family origins in Granada territory (300 km far away from that town). The three Y-C harbored the J2a1-M172 haplogroup with two of them sharing indeed 17 loci Y-STRs haplotype. These results suggest that the sampled “*vc”* males would be descendants of a member of that Muslim garrison, revealing then a clarifying case of Arab ancestry.

Other examples do reference to “appearance” surnames, such as “*mo”* (dark-haired man) and “*rb”* (blond, fair-haired man) both reaching moderate frequencies in Spain. The former registers six occurrences in the sample and all of them are linked to R-M269 lineage; the latter surname “*rb*” presents three occurrences and each Y-chromosome carries different haplogroups, being again the R-M269 one of them. It seems striking that a Mediterranean population as the Spanish is, mostly characterized by people with hair dark or dark-brown, the “*mo”* surname is less genetically diverse than those carrying “*rb”* surname. However, when the great majority of individuals within a population is well-defined by a certain phenotypic physical feature only people belonging to the extremes of the trait distribution can be successfully distinguished, and these outliers might be more genetically homogeneous and supposedly denoted with the same surname. On the contrary, the portion of the population which does not carry the common trait would be composed by people carrying the complementary trait in several degrees and forming then a more heterogeneous genetic group.

Larger, but economically feasible sample sizes than that analyzed here would not substantially increase the scope and reliability provided by the present study, due to the great disparity between the amounts of donors (sample size) and population surnames. The average number of alleles *k* (*surnames*) in a sample of size *n* in equilibrium, for large *n*, is asymptotically *θ* ln *n* [46] (although a better approximation is *k* = *θ* ln(1+*n*/*θ*) [47]). Using the former formula, when the sample size is increased 2-fold, that is, from 416 (our sample) to 832, the expected different surnames increases from 222 (our sample) to 248. In the same way, when first and second sampled surnames are combined the corresponding figures are 353 different surnames, and 124, the number of surnames with two or more occurrences. Both numbers are very small in comparison to the number of recorded surnames, and the observed range of surname frequencies in the population as well as the corresponding relationships with Y-C lineages would be quite similar in both cases.

By comparing observed surname distributions in the sample against the population where the sample was extracted, it is possible to infer whether that sample is biased or not. This comparison must be a routine for the study of human populations. Surname distributions in populations of most countries are known from national population censuses or from other data sources such as telephone and/or poll lists. Although neutrality tests cannot be performed on these huge data sets—hundreds of thousands- of surnames (alleles) (e.g. Ewens-Watterson test is currently limited to 1000 different alleles, ARLEQUIN program v. 3.5) almost surely national surname distributions would not fit well the IAM, due to similar causes than those acting on our study sample. However, a chi-square test between sampled and those all recorded population surnames is *a priori* feasible though it would not provide probably a significant difference due to the very high value of the chi-square limit. The number of different surnames fluctuates considerably due to the birth and death process (differential survival rates) governing usually singletons and quasi-singletons, and this number is difficult to define [47]. Some statistical tests between parameters, such as the exponent of power-law functions, from sample and population surname distributions may be devised in the near future, although the distribution models of these parameters are unknown. Obviously, samples where commoner population surnames are poorly represented or with surprisingly high frequencies of rare surnames would be indicating that data are biased. Similarly, a bias would be expected when sample surname distributions do not fit well enough to a power-law function or when its exponent is out of the range from -1.5 to -2. A close similitude of random inbreeding between the sample and the population is another signal of an unbiased sample.

As far as we know, it is the first time that a joint study of surnames and Y-chromosome markers in a representative population sample has been performed, at least in Spain. Other surveys [1,2,22] used less surnames (~70%) although examining many more individuals, of the order of 4 to 7 times, than that analyzed in the present work. Sampled surname frequencies are very different when analyzing British and Irish populations. For example, Irish population frequencies for Murphy, Kelly and McGuiness surnames are 1.2%, 1.2% and 0.1% respectively while the corresponding proportions in the studied samples were 5.9%, 4.9% and 8.8%. In the British case, surnames such as Smith, King and Jobling whose population frequencies are 1.3%, 0.2% and 0.006% are found in the selected samples with frequencies of 3.4%, 1.4% and 2.8% respectively. Furthermore, the inconsistent transformation of Irish surnames to English language as well as the very high frequency of Y-C R1b3 haplogroup in Ireland [48,49] constitutes a strong shortcoming for the analysis of correlations between Irish paternal lineages and surnames.

In summary, it should be important to notice that the reliability of the isonymy method should be questioned for the high rate of polyphyletic surnames, even in small geographical regions and autochthonous populations. When isonymy is used for evaluating human inbreeding, estimated values are consistently dependent of the most common surname frequencies in the population. The low correlation observed in Andalusia between surnames with different population frequencies and Y-chromosome lineages, at different levels of genetic resolution, would be mainly explained by the very low rate of monophyletic surnames, because of the historical process of surname ascription. Both conclusions might be confidently extended to other countries in the world.

## Supporting Information

S1 Table Repeated first surnames with their frequencies and haplogroup and haplotype diversities in the Andalusian sample. (DOCX)
