# Supplementary material for: Surnames and Y-Chromosomal Markers Reveal Low Relationships in Southern Spain
Source: PLoS One. 2015 Apr 10;10(4):e0123098. doi: 10.1371/journal.pone.0123098 (PMC4393112; doi:10.1371/journal.pone.0123098)
Supplement: S1 Table — (DOCX) [file pone.0123098.s001.docx]

| ***First Surname  Acronyms*** | ***Number of Individuals per Surname*** | ***Frequency (%)*** | ***Frequency (%).  Weighted average in the two selected provinces*** | ***Y-Chromosome Hg Diversity*** | ***Y-Chromosome Ht Diversity  (7 Y-STR loci)*** | ***Y-Chromosome Ht Diversity (17 Y-STR loci)*** |
| --- | --- | --- | --- | --- | --- | --- |
| *gc* | 21 | 5.048 | 4.348 | 0.6714 ± 0.1095 | 0.9895 ± 0.0193 | 0.9947 ± 0.0178 |
| *pz* | 14 | 3.365 | 2.741 | 0.7912 ± 0.0894 | 0.9670 ± 0.0437 | 1.0000 ± 0.0270 |
| *gm* | 13 | 3.125 | 1.766 | 0.7179 ± 0.1279 | 0.9697 ± 0.0443 | 0.9697 ± 0.0443 |
| *mn* | 11 | 2.644 | 2.738 | 0.8545 ± 0.0852 | 0.9818 ± 0.0463 | 1.0000 ± 0.0388 |
| *rd* | 11 | 2.644 | 3.931 | 0.4909 ± 0.1754 | 0.9818 ± 0.0463 | 0.9818 ± 0.0463 |
| *gz* | 10 | 2.404 | 2.515 | 0.7556 ± 0.1295 | 0.9778 ± 0.0540 | 1.0000 ± 0.0447 |
| *sa* | 8 | 1.923 | 2.777 | 0.7500 ± 0.1391 | 0.9643 ± 0.0772 | 1.0000 ± 0.0625 |
| *fe* | 6 | 1.442 | 3.338 | 0.3333 ± 0.2152 | 1.0000 ± 0.0962 | 1.0000 ± 0.0962 |
| *mz* | 6 | 1.442 | 2.276 | 0.6000 ± 0.2152 | 0.9333 ± 0.1217 | 0.9333 ± 0.1217 |
| *mo* | 6 | 1.442 | 1.407 | 0.0000 ± 0.0000 | 0.9333 ± 0.1217 | 0.9333 ± 0.1217 |
| *ji* | 5 | 1.202 | 1.933 | 0.4000 ± 0.2373 | 1.0000 ± 0.1768 | 1.0000 ± 0.1768 |
| *my* | 5 | 1.202 | 0.282 | 0.0000 ± 0.0000 | 1.0000 ± 0.1265 | 1.0000 ± 0.1265 |
| *vq* | 5 | 1.202 | 0.650 | 0.4000 ± 0.2373 | 1.0000 ± 0.1265 | 1.0000 ± 0.1265 |
| *do* | 4 | 0.962 | 0.912 | 0.8333 ± 0.2224 | 1.0000 ± 0.1768 | 1.0000 ± 0.1768 |
| *gv* | 4 | 0.962 | 0.213 | 0.5000 ± 0.2652 | 1.0000 ± 0.1768 | 1.0000 ± 0.1768 |
| *lp* | 4 | 0.962 | 3.214 | 0.8333 ± 0.2224 | 0.8333 ± 0.2224 | 1.0000 ± 0.1768 |
| *mu* | 4 | 0.962 | 0.986 | 0.8333 ± 0.2224 | 1.0000 ± 0.1768 | 1.0000 ± 0.1768 |
| *rz* | 4 | 0.962 | 0.512 | 0.5000 ± 0.2652 | 1.0000 ± 0.2722 | 1.0000 ± 0.2722 |
| *ro* | 4 | 0.962 | 1.263 | 0.8333 ± 0.2224 | 1.0000 ± 0.1768 | 1.0000 ± 0.1768 |
| *se* | 4 | 0.962 | 0.352 | 0.0000 ± 0.0000 | 0.5000 ± 0.2652 | 0.8333 ± 0.2224 |
| *al* | 3 | 0.721 | 0.648 | 1.0000 ± 0.2722 | 1.0000 ± 0.5000 | 1.0000 ± 0.5000 |
| *ar* | 3 | 0.721 | 0.107 | 0.0000 ± 0.0000 | 0.6667 ± 0.3143 | 0.6667 ± 0.3143 |
| *cr* | 3 | 0.721 | 0.152 | 0.6667 ± 0.3143 | 1.0000 ± 0.2722 | 1.0000 ± 0.2722 |
| *gl* | 3 | 0.721 | 0.135 | 0.6667 ± 0.3143 | 1.0000 ± 0.2722 | 1.0000 ± 0.2722 |
| *gr* | 3 | 0.721 | 0.448 | 0.0000 ± 0.0000 | 0.6667 ± 0.3143 | 0.6667 ± 0.3143 |
| *ib* | 3 | 0.721 | 0.163 | 0.0000 ± 0.0000 | 0.6667 ± 0.3143 | 0.6667 ± 0.3143 |
| *lz* | 3 | 0.721 | 0.355 | 0.0000 ± 0.0000 | 0.6667 ± 0.3143 | 0.6667 ± 0.3143 |
| *lq* | 3 | 0.721 | 0.109 | 1.0000 ± 0.2722 | 0.6667 ± 0.3143 | 0.6667 ± 0.3143 |
| *mc* | 3 | 0.721 | 0.250 | 0.0000 ± 0.0000 | 1.0000 ± 0.2722 | 1.0000 ± 0.2722 |
| *mq* | 3 | 0.721 | 0.636 | 0.0000 ± 0.0000 | 1.0000 ± 0.2722 | 1.0000 ± 0.2722 |
| *ml* | 3 | 0.721 | 0.861 | 0.6667 ± 0.3143 | 1.0000 ± 0.2722 | 1.0000 ± 0.2722 |
| *mr* | 3 | 0.721 | 0.537 | 0.6667 ± 0.3143 | 1.0000 ± 0.2722 | 1.0000 ± 0.2722 |
| *nv* | 3 | 0.721 | 0.552 | 0.6667 ± 0.3143 | 1.0000 ± 0.2722 | 1.0000 ± 0.2722 |
| *ng* | 3 | 0.721 | 0.045 | 0.6667 ± 0.3143 | 0.6667 ± 0.3143 | 1.0000 ± 0.2722 |
| *oz* | 3 | 0.721 | 0.410 | 0.0000 ± 0.0000 | 1.0000 ± 0.2722 | 1.0000 ± 0.2722 |
| *pg* | 3 | 0.721 | 0.060 | 0.6667 ± 0.3143 | 0.0000 ± 0.0000 | 0.6667 ± 0.3143 |
| *rs* | 3 | 0.721 | 0.487 | 0.0000 ± 0.0000 | 0.6667 ± 0.3143 | 1.0000 ± 0.2722 |
| *rb* | 3 | 0.721 | 0.200 | 1.0000 ± 0.2722 | 0.6667 ± 0.3143 | 1.0000 ± 0.2722 |
| *ru* | 3 | 0.721 | 1.745 | 0.0000 ± 0.0000 | 1.0000 ± 0.2722 | 1.0000 ± 0.2722 |
| *so* | 3 | 0.721 | 0.100 | 0.0000 ± 0.0000 | 0.6667 ± 0.3143 | 0.6667 ± 0.3143 |
| *vl* | 3 | 0.721 | 0.115 | 0.0000 ± 0.0000 | 0.6667 ± 0.3143 | 0.6667 ± 0.3143 |
| *vc* | 3 | 0.721 | 0.237 | 0.0000 ± 0.0000 | 0.6667 ± 0.3143 | 0.6667 ± 0.3143 |
| *av* | 2 | 0.481 | 0.181 | 1.0000 ± 0.5000 | 1.0000 ± 0.5000 | 1.0000 ± 0.5000 |
| *bb* | 2 | 0.481 | 0.045 | 1.0000 ± 0.5000 | 1.0000 ± 0.5000 | 1.0000 ± 0.5000 |
| *br* | 2 | 0.481 | 0.120 | 1.0000 ± 0.5000 | 1.0000 ± 0.5000 | 1.0000 ± 0.5000 |
| *bt* | 2 | 0.481 | 0.120 | 1.0000 ± 0.5000 | 1.0000 ± 0.5000 | 1.0000 ± 0.5000 |
| *cl* | 2 | 0.481 | 0.148 | 0.0000 ± 0.0000 | 1.0000 ± 0.5000 | 1.0000 ± 0.5000 |
| *cm* | 2 | 0.481 | 0.274 | 1.0000 ± 0.5000 | 1.0000 ± 0.5000 | 1.0000 ± 0.5000 |
| *ct* | 2 | 0.481 | 0.496 | 0.0000 ± 0.0000 | 1.0000 ± 0.5000 | 1.0000 ± 0.5000 |
| *cj* | 2 | 0.481 | 0.015 | 0.0000 ± 0.0000 | 1.0000 ± 0.5000 | 1.0000 ± 0.5000 |
| *cz* | 2 | 0.481 | 0.296 | 1.0000 ± 0.5000 | 1.0000 ± 0.5000 | 1.0000 ± 0.5000 |
| *cv* | 2 | 0.481 | 0.041 | 0.0000 ± 0.0000 | 1.0000 ± 0.5000 | 1.0000 ± 0.5000 |
| *dz* | 2 | 0.481 | 1.084 | 1.0000 ± 0.5000 | 1.0000 ± 0.5000 | 1.0000 ± 0.5000 |
| *ec* | 2 | 0.481 | 0.132 | 0.0000 ± 0.0000 | 1.0000 ± 0.5000 | 1.0000 ± 0.5000 |
| *et* | 2 | 0.481 | 0.180 | 1.0000 ± 0.5000 | 1.0000 ± 0.5000 | 1.0000 ± 0.5000 |
| *hd* | 2 | 0.481 | 0.244 | 0.0000 ± 0.0000 | 1.0000 ± 0.5000 | 1.0000 ± 0.5000 |
| *mi* | 2 | 0.481 | 0.066 | 0.0000 ± 0.0000 | 0.0000 ± 0.0000 | 1.0000 ± 0.5000 |
| *og* | 2 | 0.481 | 0.652 | 0.0000 ± 0.0000 | 1.0000 ± 0.5000 | 1.0000 ± 0.5000 |
| *rv* | 2 | 0.481 | 0.126 | 0.0000 ± 0.0000 | 1.0000 ± 0.5000 | 1.0000 ± 0.5000 |
| *ra* | 2 | 0.481 | 0.138 | 0.0000 ± 0.0000 | 1.0000 ± 0.5000 | 1.0000 ± 0.5000 |
| *ti* | 2 | 0.481 | 0.059 | 0.0000 ± 0.0000 | 0.0000 ± 0.0000 | 0.0000 ± 0.0000 |
| *vr* | 2 | 0.481 | 0.128 | 1.0000 ± 0.5000 | - | - |
| *vm* | 2 | 0.481 | 0.003 | 0.0000 ± 0.0000 | 1.0000 ± 0.5000 | 1.0000 ± 0.5000 |
